# A methodological proof-of-concept of a data-driven, personalized, blended digital health intervention for suicidal thoughts and behaviors: A case series

**DOI:** 10.1016/j.invent.2026.100917

**Published:** 2026-02-16

**Authors:** Kevin S. Kuehn, Lindsey S. Aguilar, Katherine T. Foster, Raeanne C. Moore, Colin A. Depp

**Affiliations:** a University of California San Diego, Department of Psychiatry, United States; b University of Washington, Department of Psychology, United States; c University of Washington, Department of Global Health, United States

**Keywords:** Suicide prevention, Idiographic modeling, Ecological momentary assessment (EMA), Digital mental health, Dialectical behavior therapy (DBT), Personalized intervention

## Abstract

**Introduction:**

Suicidal thoughts and behaviors (STBs) are a leading cause of death in the United States. Individuals at high-risk for suicide vary greatly in their precedents to STBs, which hinders suicide prevention strategies. Personalized approaches to mapping individualized precedents to suicide ideation might increase the impact and efficiency of treatment.

**Methods:**

The present study describes a personalized, blended digital health treatment that uses idiographic network models derived from ecological momentary assessment to inform treatment targets (PeRsonalizEd Clinical Intervention for Suicide Events; PRECISE). PRECISE includes skills from dialectical behavior therapy and safety planning, two existing evidence-based treatments. In this case series, participants (*N* = 5) at high-risk for suicide and completed a 6-week treatment which included 5×/day ecological momentary assessments as well as weekly coaching sessions. Outcomes were assessed at baseline, post-treatment, and six-weeks post-treatment.

**Results:**

In the intent-to treat sample, three of the five participants (60%) completed the full treatment protocol. Participants attended an average of 4.4 coaching sessions (73.3%), adherence was excellent (98%), and satisfaction was also high (4.2 out of 5). The severity of suicidal thoughts and behaviors were reduced at both post-treatment and the 6-week follow-up (d_zs_ = −1.33 to −2.00).

**Conclusions:**

PRECISE is an example of a blended digital health interventions that capitalizes on time series data to personalize interventions for suicidal thoughts and behaviors. Incorporating real-time data and idiographic models to inform clinical decision making are promising tools to improve suicide care. Lessons learned and future directions for implementation are discussed.

## Highlights

• PRECISE is a personalized, blended digital intervention for suicidal ideation.
• Idiographic EMA networks were used to tailor treatment targets in real time.
• The 6-week case series showed high engagement and satisfaction with PRECISE.
• Suicidal thoughts decreased substantially from baseline to follow-up (dz = −1.33 to −2.00).
• PRECISE demonstrates feasibility and promise for personalized suicide prevention.

## 1 Introduction

Suicide, or intentional self-injury resulting in death, is a leading cause of mortality in the United States. Over 49,000 people died by suicide in 2023 (Centers for Disease Control and Prevention, 2024). Suicidal thoughts and behaviors (STBs) are more common with an estimated 12.8 million individuals (4%) reporting that they seriously contemplating suicide in the past year, while approximately 1.5 million (<0.01%) individuals reported they attempted suicide in the same time span. Suicidal thoughts and behaviors can be a precursor to future suicide attempts and death (Ribeiro et al., 2016).

Despite many efforts at preventing suicide, the suicide rate has continued to increase since 2000 (Centers for Disease Control and Prevention, 2024). One of the cornerstones of indicated suicide prevention is suicide-specific psychosocial interventions. Notable interventions include cognitive-behavioral therapy for suicide prevention (CBT-SP) and dialectical behavior therapy (DBT). DBT has been extensively studied with 18 randomized controlled trials showing reductions in self-injurious behavior (DeCou et al., 2019). Originally intended for individuals at high-risk for suicide, DBT has been used mainly to treat women meeting criteria for borderline personality disorder – a group at elevated risk for suicide (Linehan et al., 2006). Emerging evidence suggests that the skills training component of DBT may be the most effective module of the treatment in reducing self-injurious behaviors (Linehan, 2015), but the mechanisms that contribute to *individual-level* change remain unclear.

DBT may be particularly effective because both theoretical and empirical evidence suggest that its group-level mechanism of action involves replacing maladaptive behaviors, such as suicidal thoughts and behaviors (STBs), with more effective emotion regulation skills (Lynch et al., 2006, Neacsiu et al., 2010). Indeed, evidence from observational studies highlights the importance of affect regulation in maintaining STBs (Kuehn et al., 2022a). Furthermore, a systematic review of imaging studies reported that deficits in medial and lateral ventral medial prefrontal cortex, along with the connections between them, were associated with increased negative and reduced positive emotions as well as suicidal thoughts. Thus, deficits in the dorsal prefrontal cortex and inferior frontal gyrus system, important for affect regulation and impulse control, may explain the transition from suicidal thoughts to suicidal behaviors (Schmaal et al., 2020). This is consistent with the affect regulation function of suicidal thoughts and behaviors (Kuehn et al., 2022a), which posits that individuals likely engage in STBs as a form of maladaptive emotion regulation to reduce distressing emotions. Across instances of this negative reinforcement pathway, the likelihood of STBs continues increasing, further increasing the need for skills that may be deployed flexibly across time and situations to manage and reduce distress via other strategies. Consequently, psychosocial interventions that aim to replace maladaptive emotion regulation strategies (e.g., STBs) with more effective emotion regulation strategies that can be engaged through an agile repertoire during moments of distress would likely support decreases in STBs over time.

Considering that STBs are complex and multiply determined (Kuehn et al., 2019; Franklin et al., 2017), researchers have recently started to take advantage of the ubiquitous adoption of smartphone technology to study STBs as they occur in peoples' daily lives. Ecological momentary assessment (EMA), or mini surveys delivered multiple times per day, is the most common approach to capturing many observations per person over time. The temporal richness of EMA data has specific advantages for disaggregating between and within person variance. Specifically, inferences can be expanded from exclusive focus on differentiating between individuals at high versus low risk to allowing for more precise specification of *when* someone is at elevated risk or how STBs change over time within a specific individual. However, models used for disaggregation often examine within-person factors with a group-level average (i.e., nomothetic), which assumes findings apply to all participants. Prior studies have demonstrated that EMA data can be non-ergodic (e.g., probabilities of suicidal thinking differ across individuals), violating an important assumption that both between and within-person average effects generalize to all individuals. To the extent that EMA data are non-ergodic, individual-level (i.e., idiographic) methods can have unique advantages for building insights about person-specific processes.

Interventions designed to leverage the potential benefits of personalization have been evaluated in the treatment of other mental health conditions. In these interventions, the primary means of personalization has been to gather individual level data through EMA and use idiographic techniques to identify treatment targets unique to the individual in conditions such as eating and anxiety disorders (Levinson et al., 2021; Levinson et al., 2023; Fisher et al., 2019). These personalized interventions have the potential to be more efficient than manualized treatments as they can tailor interventions more closely to person-specific determinants of clinical problems across individuals and time, reducing cost and saving time. This may be especially relevant to suicide prevention as existing evidence-based approaches to STBs (e.g., DBT) are poorly disseminated (Jobes and Barnett, 2024).

We developed a new idiographic blended intervention for young adults at high risk for suicide, called PRECISE. We targeted this population because suicide risk often first emerges during young adulthood (Mortier et al., 2017), presenting a critical window for prevention efforts. PRECISE advances the field of digital mental health by utilizing automated, real-time generation of idiographic network models to trigger specific clinician-led skill selection. Unlike prior digital adaptations of DBT that rely on static manuals or simple mood-tracking, this data-driven framework identifies a patient's unique affective precursors to inform personalized treatment targets. To our knowledge, there are no prior reports of personalized interventions for individuals experiencing suicidal thoughts and behaviors.

The current study presents a case series analysis aimed at informing the further refinement of the PRECISE intervention. The aims of the present study are to: 1) describe a data-driven personalized intervention for suicide risk and 2) present a preliminary case series analyses of individuals who have received the personalized intervention and 3) introduce a pilot open trial of PRECISE. It was hypothesized that the individuals who received the personalized intervention would find it to be feasible, and acceptable, and that engagement in the intervention would be linked to reductions in the intensity of their negative emotions, increases in effective emotion regulation strategy use, and decreases in the severity of their suicidal thoughts and behaviors.

## 2 Methods

### 2.1 Study design and overview

The present study was an uncontrolled, open trial to develop the manual of a personalized intervention and the procedures for a larger pilot study. Five participants were targeted for the first phase of a two-phase project. The trial included assessments at baseline, six-weeks (post-treatment), and 12-weeks (6-weeks post-treatment). The coaching sessions lasted 6 weeks in which participants met with a coach once per week and completed 5×/day EMAs between sessions.

### 2.2 Participants

Inclusion criteria included: 1) 18–26 years old 2) endorsement of active suicidal ideation on the Columbia Suicide Severity Rating Scale (CSSRS; Posner et al., 2011; >2 or past months thoughts of killing self) 3) English fluency, and 4) willingness to provide contact information for a key information to be contacted as part of our risk and safety plan. Exclusion criteria included: 1) Unable to read in English, and 2) a diagnosis of psychotic or severe thought disorder (e.g., schizophrenia, bipolar disorder with psychotic features).

Participants were mostly recruited from online social media sources as well as through referrals from local college mental health clinics. Potential participants (*N* = 50) first completed a brief online screen. Eligible participants (*N* = 29; 58%) were then contacted to complete a brief one-hour screen via virtual teleconference software. All procedures were approved by the Institutional Review Board at the University of California San Diego (protocol #806080).

### 2.3 Procedures

Participants that continued to be eligible were scheduled for an in-person baseline assessment (*N* = 9; 15 participants did not respond to requests to schedule a baseline, and 5 participants lost interest in participating following online screen). Those maintaining eligibility were scheduled for their initial coaching session (*N* = 5; 4 participants were determined ineligible following baseline). During this baseline appointment, participants were enrolled in the EMA platform by providing their email address and cell phone number, completed guided practice with EMA items, and scheduled their EMA signal-contingent windows. Participants were asked to complete both “signal-contingent” prompts (i.e., 5×/day surveys delivered during randomly generated scheduling windows) and “event-contingent” surveys (i.e., self-initiated surveys completed when experiencing self-injurious thoughts/behaviors outside of signal-contingent windows). Participants were compensated $30 for the baseline appointment and $15 for each of the two follow-up assessments (6-week, and 12-week). They were also compensated $0.50/signal-contingent survey with a $25 bonus if they completed >80% of the EMAs. Participants were not compensated for event-contingent surveys.

During the study, participants completed the following in-person and ecological momentary assessments:

#### 2.3.1 In-person assessments

##### 2.3.1.1 Primary outcome

Columbia Suicide Severity Rating Scale (C-SSRS; Posner et al., 2011): The C-SSRS is a 28 item semi-structured interview that assesses the frequency, intensity, duration, and function of lifetime and past-month suicidal ideation. The C-SSRS also classifies suicidal behaviors based on categories in line with CDC recommendations. Specifically, actual suicide attempts, or behavior with the intent to kill oneself, is differentiated from preparatory, aborted, and interrupted suicide attempts. Intensity of suicidal ideation and suicidal behaviors were assessed at baseline (past-month and lifetime), 6-weeks (post-treatment; past-month), and 12-weeks (6-weeks post-treatment; past-month).

##### 2.3.1.2 Secondary outcome

Modified Scale for Suicide Ideation (MSSI: Miller et al., 1986): The MSSI is an 18-item semi-structured interview that assess the frequency, intensity, and duration of suicidal ideation in the past 48 h. Items on the MSSI range from 0 (none/absent) to 3 (high) and are summed to create a suicidal ideation severity score in the past 48 h (range = 0 to 54).

##### 2.3.1.3 Satisfaction questionnaire

The System Usability Scale (SUS; Brooke, 1996) is a ten item Likert scale self-report measure designed to evaluate the effectiveness, efficiency, and satisfaction of a digital system. Items on the SUS range from 1 (“strongly disagree”) to 5 (“strongly agree”). Odd numbered responses are subtracted by 1, while even numbered responses are subtracted by five. The converted scores are then summed, which is multiplied by 2.5 to create a composite score that ranges from 0 to 100. Scores above 68 are generally considered “above average”.

##### 2.3.1.4 Acceptability and feasibility

The Acceptability of Intervention Measure, Feasibility of Intervention Measure, and Intervention Appropriateness Measure (AIM/FIM/IAM; Weiner et al., 2017) was adapted to measure the acceptability, feasibility and appropriateness of the personalized intervention. The AIM/FIM/IAM is a 12-item measure with responses ranging from 1 (“completely disagree”) to 5 (“completely agree”). Items derived from the various subscales (e.g., acceptability, feasibility) were averaged to create a mean score.

#### 2.3.2 EMAs (5×/day for 42 days)

##### 2.3.2.1 Negative and positive emotions

Participants rated the intensity of their positive and negative emotions *in the past 10* min using a 0–100 drop down box. Specific negative emotions included sadness, fear, anger, guilt, shame while positive emotion items included (joy, love, calm, attentive, confident). The items were adapted from the Positive Affect - Negative Affect Scale (PANAS; Watson et al., 1988) and adapted to be relevant to DBT-based emotion regulation modules.

##### 2.3.2.2 Emotion regulation strategies

Participants were asked whether they engaged in any of the following adaptive and maladaptive emotion regulation strategies during each signal- and event-contingent EMA: 1) cognitive reappraisal; 2) rumination; 3) acceptance; 4) distraction; 5) avoidance; 6) problem-solving; 7) suppression; 8) self-invalidation. These items were adapted from the Cognitive Emotion Regulation Questionnaire (CERQ; Garnefski and Kraaij, 2006). Maladaptive strategies, which included rumination, distraction, avoidance, suppression and self-invalidation were summed to create a composite score of maladaptive strategy use at each EMA observation.

##### 2.3.2.3 STBs

Participants were asked whether they experienced any self-injurious thoughts and behaviors in both the signal- and event-contingent EMAs. An initial gate-keeping question asked participants whether they have “thought about hurting or killing themself” since the last assessment. If participants answered “yes”, they were then asked about their intention to harm themselves in the past 30 min [1 - “None” to 5 - “Severe - I harmed myself”], their intention to kill themselves in the past 30 min [1 - “None” to 5 - “Severe - I thought about and had a specific plan and/or started taking action”], and whether they engaged in any non-suicidal or suicidal self-injurious behavior since the last assessment. If participants reported any self-injurious behavior, they were asked about the method, severity, function, and use of emergency and/or social support resources.

##### 2.3.2.4 PRECISE

Participants completed a total of six manualized coaching sessions conducted either through HIPAA-compliant virtual teleconferencing software or in-person. These coaching sessions all followed a similar format with 1) review of EMA data from the past week and idiographic networks 2) suicide risk assessment 3) teaching, reinforcing, and strengthening learning of DBT-based skills and 4) completion/review/update of their safety plan.

The first two sessions of PRECISE were standardized and not tailored based on EMA data to allow for a “learning period” and to increase the within-person power of idiographic networks. Standardized procedures for these first two sessions involved orienting participants to the DBT-based skills and teaching generic emotion regulation related content (e.g., function of emotions, describing and differentiating emotions, cognitive interpretations). Starting in session 3, treatment was then individually tailored using a combination of EMA data, chain analysis, and clinical judgment. These latter sessions followed a similar format to the first two in which coaches reviewed data from the past week, conducted a suicide risk assessment, taught individualized DBT-based skills, and updated participants' safety plans (see Table 3 for more information on the specific DBT skills taught in PRECISE).

##### 2.3.2.5 Analysis and integration of network models

**Fig. 1:**
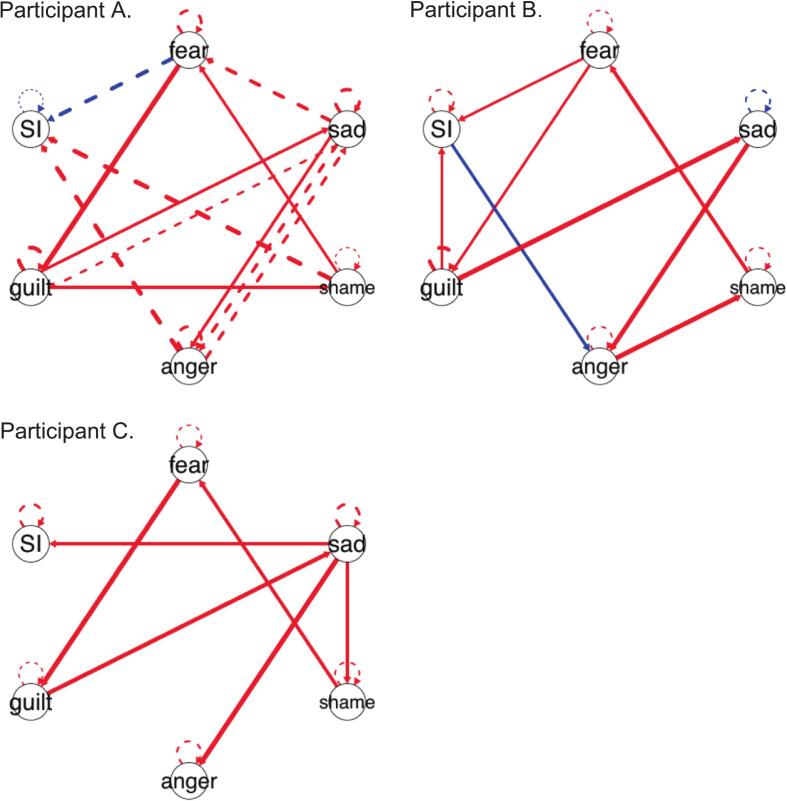
Idiographic networks for treatment completers. *Notes:* Nodes represent momentary variables (e.g., Sadness, SI) measured via EMA. Edges (lines) represent the temporal relationship between variables (dotted = lagged, solid = contemporaneous). Red edges indicate a positive predictive relationship (e.g., an increase in shame predicts an increase in SI), while blue edges indicate an inhibitory relationship. Line thickness represents the strength of the association (*beta* weight). Arrows indicate directionality based on vector autoregression (VAR) models.

Group and idiographic network models (Fig. 1**)** were generated using GIMME (Lane et al., 2024) in R Studio (RStudio Team, 2024, version 2023.12.1.402). Based on structural equation modeling and vector autoregression, GIMME derives both nomothetic, or group-level, and person-specific network models. EMA data was downloaded every 24 h, and scripts were created to process data such that individual participant files were split into separate text files (“.txt”) and processed through the GIMME R script. Individual output graphs were then added to the participant dashboard.

##### 2.3.2.6 Idiographic patient dashboard (Fig. 2)

A real-time R Shiny dashboard was created in which EMA data was downloaded every 24 h and displayed individual trajectories of suicidal thoughts and behaviors over time. The coach facing dashboard also listed timestamps for each STB, the correlations between negative emotions and suicidal thoughts, trajectories of negative emotions over time, frequencies of emotion regulation strategies, and person-specific network models. Each person's data was reviewed with them at the beginning of each session and used to inform which DBT skills were taught during the session.

**Fig. 2:**
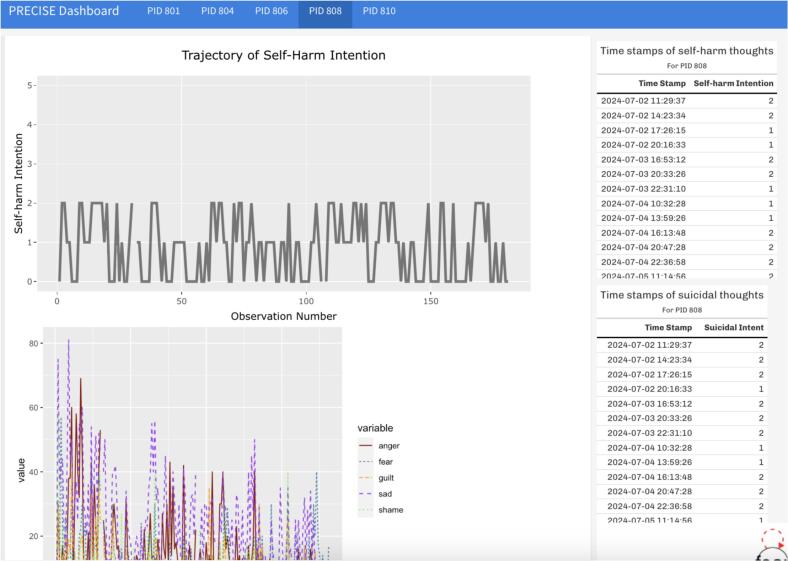
Coach facing dashboard. *Note:* The dashboard visualizes real-time EMA data. The top panel shows the longitudinal trajectory of suicidal thoughts (0–100 intensity) across the 6-week intervention. The lower panel displays igiographic network models, allowing the coach to pivot the session focus based on the previous week's most influential emotional “hubs.”

##### 2.3.2.7 Tailoring procedures

Individual DBT skills were delivered to participants based on a combination of the idiographic network models and a behavioral chain analysis. Based on preliminary data (Kuehn et al., 2024), three exemplary idiographic network models were identified across participants at high-risk of suicide. For one group, a single emotion was correlated with suicidal thoughts, while another group had multiple emotions correlated to suicidal thoughts. A third group did not have any affective links with suicidal thoughts. Based on these groups, different DBT modules were identified to target suicidal thoughts. For example, if a participant's network identified a link between a specific emotion (e.g., fear) and a suicidal thought, a coach would guide the participant through emotion regulation strategies tailored to that emotion (e.g., using “check-the-facts” or “opposite-to-fear” actions for fear). However, if a participant's network revealed multiple emotional links to suicidal thoughts, the coach would instead focus on distress tolerance strategies (e.g., using “STOP,” urge surfing, or other crisis survival skills). At the beginning of each session, the coach confirmed the accuracy of the network model through a behavioral chain analysis which was then used to focus on specific DBT strategies for different specific emotions.

##### 2.3.2.8 Monitoring and response to suicidal thoughts in EMA

EMA responses were monitored daily, and study staff received an alert when participants reported data indicating imminent risk of suicide. Imminent risk was defined as greater than a 4 on the item assessing their current intention to die by suicide and/or if a participant reported attempted suicide (defined as a self-injurious behavior with intention to die). Study staff would then attempt to reach participants to complete a risk assessment and take steps to mitigate risk and/or reach out to emergency contacts if a participant could not be reached within 24 h. All participants needed to provide contact information for an emergency contact to participate in the study. There was only one observation in which a participant reported a ‘4’ on their current intention to die by suicide.

##### 2.3.2.9 Data analysis

Within-subjects effect sizes (Cohen's d) were calculated based on means and standard deviations. Within subject linear models were used to examine individual trajectories in suicidal thoughts as well as negative emotions for each participant. Raw data, processing and analysis scripts, and other materials are available at https://github.com/kskuehn/PRECISE-case-series.

## 3 Results

### 3.1 Feasibility and engagement

See Table 1 for clinical and demographic characteristics of the ITT sample. On average, participants (M_age_ = 21.2; 40% White, 20% Asian, 20% Black, 20% mixed ethnicity, 80% female, 100% cis-gendered) completed 85.68% of the signal-contingent EMAs. There were seven additional self-initiated (“event-contingent”) surveys completed by one participant. Additionally, the three participants who completed treatment attended 100% of the six coaching sessions. The two participants who did not complete treatment either withdrew (*n* = 1) or were lost-to-follow-up (n = 1), resulting in a 40% attrition rate. Both instances occurred during the second week of the study participation. The ITT sample attended an average of 4.4 sessions (73.3% of the intended sessions). The mean adherence score was 98.9% (range = 0–100%).

**Table 1:** Demographic and clinical characteristics.

Variable	M (SD)/N (%)
Age	21.2 (1.92) (Range = 19–22)
Sex	
Female	4 (80%)
Male	1 (20%)
Race/Ethnicity	
White	2 (40%)
Asian	1 (20%)
Black	1 (20%)
Mixed race	1 (20%)
Self-reported mental health diagnosis	
Depression	3 (60%)
Anxiety	1 (20%)
Post-traumatic stress disorder (PTSD)	1 (20%)

### 3.2 Clinical outcomes

Given the small sample size, inferential statistics were deemed inappropriate. Instead, we calculated within-subject effect sizes (*d*_*z*_) for treatment completers (*N* = 3) to describe the magnitude of change in suicidal ideation (C-SSRS). From baseline to post-treatment (6-weeks), a large descriptive effect size was observed (*d*_*z*_ = −2.00), which was maintained at the 12-week follow-up (*d*_*z*_ = −2.00). However, given the extremely small *N*, these values are highly sensitive to individual variance and should be interpreted as preliminary evidence of clinical trend rather than stable estimates of population efficacy. Mean acceptability (AIM) was 4.2/5, and the System Usability Scale (SUS) score was 72.5, which is categorized as “above average.” However, the feasibility of the EMA-driven algorithm was challenged by discrepancies in reporting (see Participant C) (Table 2).

**Table 2:** Pre-post changes in clinical outcomes (*n* = 3).

Variable	Baseline	6-week	12-week	d_z_
	M (SD)	M (SD)	M (SD)	
CSSRS	3.6 (0.89)	1.33 (1.15)	1.33 (1.53)	-2.00
MSSI	12 (7.52)	5 (4.36)	1.33 (1.15)	−1.33

*Note.* CSSRS = Columbia Scale for Suicide Severity, MSSI = Modified Scale for Suicidal Ideation.

**Table 3:** Specific skills taught in PRECISE coaching sessions.

Mindfulness	Emotion regulation	Distress tolerance
Observe	Check the facts	STOP
Describe	Opposite action	*Pros and Cons*
Participate	Problem-solving	TIPP
Nonjudgmentally	Accumulating positive emotions	Distract
One-mindfully	Building mastery	Self-soothe
Effectively	Coping ahead	Improve the moment
	Reducing vulnerability	Radical acceptance
	Mindfulness of current emotion	Willingness/mindfulness of current thoughts

### 3.3 Individual profiles

Individual profiles for the three participants who completed the treatment protocol are presented below.

#### 3.3.1 Participant A

##### 3.3.1.1 Background and case conceptualization

Participant A was a 19-year-old female presenting with a history of suicidal ideation and self-injurious behavior. Her suicidal ideation started about six years ago during her freshman year of high school. At baseline, Participant A reported her suicidal ideation was often triggered by perceptions that others are “thinking negatively of [her].”

##### 3.3.1.2 Early treatment EMA data

At the first coaching session, Participant A's EMA data revealed three instances of self-injurious thoughts which occurred over the course of one day. These thoughts started in the afternoon and lasted until the evening survey. Both the individual network model (Fig. 1.1) and a behavioral chain analysis revealed that participant A reported high levels of shame preceding self-injurious thoughts. During session one, the client was provided with basic psychoeducation on emotions and ways to describe emotional states. Between session one and session two, participant A reported in EMA that she engaged in self-injurious behavior via scratching. Participant A denied she had any suicidal intent and reported that she scratched herself to get rid of a thought/feeling. She also denied that she needed any medical care or reached out to others. As she continued to deny further self-injurious thoughts and behavior on her subsequent EMAs, the emergency response protocol was not activated, and this behavior was discussed at the subsequent session (session two).

##### 3.3.1.3 Use of EMA data to inform treatment plan

During session two, this information was reviewed along with a suicide risk assessment and a behavioral chain analysis surrounding the self-injurious behavior. Participant A was also introduced to cognitive reappraisal and exposure-based strategies (i.e., “opposite-to-emotion action”). As participant A reported that the emotion was “unjustified” (i.e., she did not know if others were rejecting her and did not feel that other people's opinions were important to her”), she was instructed how to engage in “opposite action” to shame. Participant A was assigned homework to practice this skill between sessions and to intentionally seek out experiences in which she was were interacting with others in ways she might perceive others rejecting her. Participant A continued to practice this skill during subsequent sessions and specific instances in which she perceived others to be evaluating her were reviewed in session.

##### 3.3.1.4 Trajectory of STBs and other clinical variables

Participant A completed seven self-initiated surveys during the first few weeks of the study, but did not report any other instances of self-injurious behavior over the duration of the study. Her EMA data revealed reductions in her self-injurious thinking across sessions (b = −0.001, OR = 0.99, t [df = 210] = −3.21, *p* = .001) and the intensity of her negative emotions (b = −0.05, t [df = 210] = −6.96, *p* < .001).

#### 3.3.2 Participant B

##### 3.3.2.1 Background and case conceptualization

Participant B was a 20-year-old female with a history of depression, anxiety, and suicidal ideation. Participant B reported that her suicidal ideation started around 14 years old at which point she reportedly attempted suicide via overdose.

##### 3.3.2.2 Early treatment EMA data

At session one, participant B's individual network model and a behavioral chain analysis revealed that participant B's suicidal thoughts were prompted by a combination of fear and shame (e.g., fear of failing in school/finding employment and shame that they are not good enough). Visual inspection of their EMA data also suggested that her suicidal thoughts increased throughout the day and were gradually increased throughout the day. Participant B was provided with basic psychoeducation on emotion and emotional states at session one.

##### 3.3.2.3 Use of EMA data to inform treatment plan

During session two, participant B was also given information on cognitive reappraisal and opposite action. As participant B's fear and shame were both “unjustified”, she was provided with tools to reevaluate the situation and to practice opposite action.

##### 3.3.2.4 Trajectory of STBs and other clinical variables

Although participant B's suicidal thinking continued to fluctuate over the course of the study, the severity of her suicidal thinking (b = −0.002, t [df = 176] = −2.58, *p* < .001) as well as the intensity of her negative emotions decreased over time (b = −0.09, t [df = 208] = −11.47, p < .001). She did not complete any self-initiated surveys.

#### 3.3.3 Participant C

##### 3.3.3.1 Background and case conceptualization

Participant C was a 22-year-old female with a history of depression, anxiety, post-traumatic stress disorder, and suicidal ideation. She reported her suicidal ideation started around 12 years old. She reported she experienced suicidal ideation intermittently since then.

##### 3.3.3.2 Early treatment EMA data

Participant C did not report any suicidal thoughts in EMA over the course of the study; however, she did verbally report to their coach that she experienced brief ideation (in sessions 2, 3, and 4). Participant C reported that these thoughts occurred outside of the random intervals, and she did not complete event-contingent surveys.

##### 3.3.3.3 Use of EMA data to inform treatment plan

Behavioral chain analyses revealed that participant C experienced suicidal thoughts due to sadness and a feeling that her life was ‘meaningless’. Sessions then focused on effective emotion regulation, accumulating positive emotions, values clarification, and increasing values directed behavior.

##### 3.3.3.4 Trajectory of STBs and other clinical variables

As participant C did not report suicidal thoughts during the treatment in EMA, the probability of suicidal thinking did not change over time, nor did the intensity of negative emotions.

## 4 Discussion

This is the first treatment to our knowledge to integrate idiographic analyses to inform personalization of treatment for self-injurious thoughts and behaviors. The initial proof of concept study suggests that this approach may be feasible, acceptable, and generally well-tolerated by participants. Pending a larger study, group-level models demonstrated reductions in negative emotions, maladaptive coping strategies, and suicidal ideation over the course of the study.

Consistent with an emerging body of research (Kuehn et al., 2022b; Coppersmith et al., 2024; Kaurin et al., 2022, Yin et al., 2023), the present study provides additional evidence of between-person heterogeneity in within-person process of suicidal thoughts. The present study also suggests that treatments which incorporate principles and skills of DBT, tailored to these unique within-person processes, can be delivered and may be effective. Although the severity of suicidal thoughts was reduced for the three participants who completed the study, all three participants continued to experience a mild level of suicidal thoughts.

A critical observation in this case series was some discrepancy between digital and verbal reporting. Participant C reported suicidal thoughts during coaching sessions that were not captured by the 5×/day EMA prompts. This disclosure gap suggests that while idiographic models provide high-resolution data, they may not yet capture the full clinical picture in acutely distressed populations. This aligns with recent scholarship emphasizing that digital and AI-driven psychotherapy must be viewed as “blended” tools rather than replacements for clinical rapport (Beg et al., 2024; Beg and Verma, 2025).

Furthermore, the presence of between-person heterogeneity in within-person linkage of affective states and suicidal thoughts and behaviors (Kuehn et al., 2024) suggests that emotion regulation skills separately uniquely across individuals. – indeed, the cases focused on emotion differentiation, cognitive re-appraisal, and opposite-action to shame and fear. Therefore, further refinement of personalized interventions such as the one in the present study is warranted. Brief, cost-effective, and personalized treatments—such as the one developed in this study which uses DBT skills to target similar mechanisms—may offer implementation advantages over full-scale DBT, which is time-consuming, costly, and less efficient.

This methodological proof-of-concept demonstrates the potential for higher efficiency in suicide-specific care, though it requires further validation in larger, controlled trials. Phase two of this project, which is an open trial (*n* = 30) in the a similar patient population, as well as an NIH-funded clinical trial in a different patient population (K23MH137367), are currently underway to evaluate the efficacy of PRECISE and to obtain implementation related outcomes (e.g., feasibility, acceptability, cost-effectiveness, etc.; Kuehn et al., 2025).

### 4.1 Limitations and future directions

PRECISE uses nomothetic and idiographic network models as well as a therapist guided behavioral chain analysis to inform the selection of specific DBT skills. EMA data was incorporated with clinical judgment as understanding emotions and the effective regulation of them is dependent on person by environment interactions that cannot be fully captured through retrospective self-report surveys/clinical interviews or passive sensing. This is because emotional states include interactions between an individuals' thoughts, environmental circumstances, and behaviors (Barrett, 2017). Thus, the EMA data is intended to serve as a prompt for increasing the reliability of behavioral chains and the network analyses allow for a coach and participant to gain insights as to temporal patterns between emotions and suicidal thoughts and behaviors. The factors that maintain suicidal thoughts and behaviors, which may differ from the etiological processes that lead to their development, are directly targeted by the therapist.

There are a few limitations to this study. Though a case series analysis is intended to showcase and allow juxtaposition of rich, nuanced details across cases, it does not constitute a generalizable test of the personalized intervention. That is, this work offers a proof-of-concept and very preliminary evidence as to the viability of data-driven personalized interventions for suicidal thoughts and behaviors. Additionally, an open trial and convenience sample was used without comparison to alternative coaching, limiting the ability to make causal inferences as to the efficacy of these types of interventions independently or relative to others. It is unclear how much of an improvement the intervention would be over treatment as usual or how suicidal thoughts and behaviors naturally change over time. The 40% attrition rate suggests that the burden of 5×/day EMA may be too high for some high-risk individuals. Furthermore, the statistical instability of effect sizes in such a small sample size warrants extreme caution in interpretation.

Future directions for this work are numerous. One line of possible research is to incorporate automation to reduce the duration of the treatment and clinician workload. Large language models, such as Chat GPT-4 (OpenAI, 2023), could be trained to allow an individual to gain an interactive understanding of their emotional dynamics and to learn personalized skills for effectively regulating them. Improvements in the ability for large language models to perform numerical reasoning as well as ways to keep these models safe for individuals at high-risk of suicide are needed to advance this line of work. Additional future directions include taking a user-centered design approach to inform how to best communicate results derived from idiographic methods to increase patient insight and maximize the utility of these methods. Additionally, questions such as which patterns of suicidal thoughts are most effectively addressed by the various intervention strategies need to be answered before the widespread use of personalized interventions. The present study focused on emotion and emotion regulation due to affect regulation models of STBs and the mechanism of action in DBT; however, other related constructs such as interpersonal functioning as well as contextual factors could be used to inform the personalization process. Future research could incorporate these constructs. It remains to be answered as to whether personalization leads to more efficient treatments and if personalization makes treatments more effective. Finally, identifying for whom personalized interventions may be most effective (as opposed to someone who responds to a manualized treatment) needs to be clearly delineated.

The integration of data-driven modeling into suicide prevention raises important questions regarding responsible AI and ethical inquiry (Beg, 2025). Our protocol required daily monitoring by doctoral-level staff, which presents a significant barrier to scalability. Future research should explore “lay-coach” models or more automated safety protocols to broaden implementation without compromising the ethical standard of care. Furthermore, the extent to which clinicians find idiographic statistical models useful in informing their treatment plans as well as their motivation to incorporate them could assist with implementing the treatment. Finally, the intervention was designed to be delivered on an outpatient basis for individuals not requiring hospitalization. This could be deployed for individuals being discharged from inpatient treatment as the rate of suicide is elevated in the month following discharge. Identifying how personalized interventions could be most effective at each level of care should be addressed, and incorporating patient preferences in these decisions could help to increase sustainability.

This study offers promising initial support for the feasibility and acceptability of a personalized DBT-informed intervention guided by idiographic models for individuals experiencing suicidal thoughts and behaviors. By integrating ecological momentary assessment, person-specific network models, and therapist-led behavioral analyses, the PRECISE intervention represents a novel and potentially impactful approach to enhancing the precision of suicide prevention efforts. While preliminary, these findings underscore the need for continued innovation in tailoring treatments to individual emotional and behavioral patterns. As the field moves toward more scalable and data-informed mental health care, future work should prioritize rigorous testing of these methods, refinement of implementation strategies across diverse settings, and identification of the individuals most likely to benefit. Personalized interventions hold considerable promise, and this study serves as an early step toward realizing their potential to more effectively and efficiently reduce the burden of suicide.

## Funding

Funding was provided by an Early Career Researcher grant from the 10.13039/100001455American Foundation for Suicide Prevention awarded to Dr. Kevin S. Kuehn (AFSP; grant number YIG-0-078-22). The 10.13039/100000002National Institutes of Health also provided support for this research (T32-AI-007384). The funders did not play any role in the design, execution, or interpretation of the study.

## Declaration of competing interest

The authors declare no conflicts of interest. No financial or personal relationships influenced the conduct, analysis, or reporting of this study.
